# Adhesive, injectable, and ROS-responsive hybrid polyvinyl alcohol (PVA) hydrogel co-delivers metformin and fibroblast growth factor 21 (FGF21) for enhanced diabetic wound repair

**DOI:** 10.3389/fbioe.2022.968078

**Published:** 2022-08-31

**Authors:** Hong Zhu, Jie Xu, Min Zhao, Hangqi Luo, Minjie Lin, Yuting Luo, Yuan Li, Huacheng He, Jiang Wu

**Affiliations:** ^1^ Department of Endocrinology, The First Affiliated Hospital of Wenzhou Medical University, Wenzhou, Zhejiang, China; ^2^ Key Laboratory of Biotechnology and Pharmaceutical Engineering, School of Pharmaceutical Sciences, Wenzhou Medical University, Wenzhou, Zhejiang, China; ^3^ College of Chemistry and Materials Engineering, Wenzhou University, Wenzhou, Zhejiang, China

**Keywords:** PVA, FGF21 (fibroblast growth factor 21), diabetic wound, ROS, hydrogels

## Abstract

As conventional treatments for diabetic wounds often fail to achieve rapid satisfactory healing, the development of effective strategies to accelerate diabetic wound repair is highly demanded. Herein, fibroblast growth factor 21 (FGF21) and metformin co-loaded multifunctional polyvinyl alcohol (PVA) hydrogel were fabricated for improved diabetic wound healing. The *in vitro* results proved that the hydrogel was adhesive and injectable, and that it could particularly scavenge reactive oxygen species (ROSs), while the *in vivo* data demonstrated that the hydrogel could promote angiogenesis by recruiting endothelial progenitor cells (EPCs) through upregulation of Ang-1. Both ROSs’ removal and EPCs’ recruitment finally resulted in enhanced diabetic wound healing. This work opens a strategy approach to diabetic wound management by combining biological macromolecules and small chemical molecules together using one promising environmental modulating drug delivery system.

## Graphical Abstract

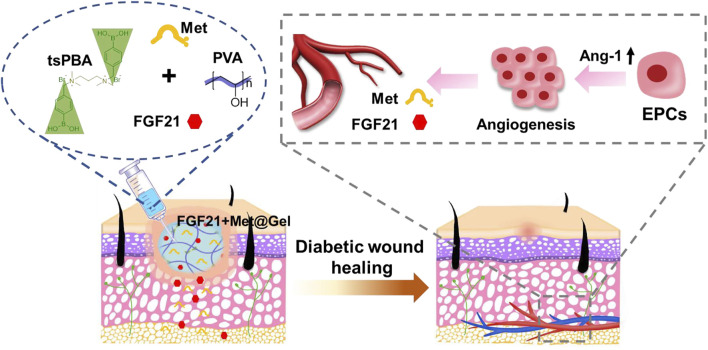

## Introduction

As the skin works as a protective shield for the body to defend against external damages, it is vulnerable to suffering from injury (Takeo et al., 2015; Xiao J. et al., 2021). Once the skin gets wounded, a healing process will be spontaneously initiated. This process includes hemostasis, inflammation, proliferation and angiogenesis, re-epithelialization, and remodeling (Demidova-Rice et al., 2012; Sorg et al., 2017; Xiao Z. et al., 2021). Under this normal process, the wound usually will be repaired within a few days/weeks (Demidova-Rice et al., 2012; Ibrahim et al., 2018). However, the wound healing process becomes abnormal in patients with diabetes mellitus, and their wounds often take longer to heal or even remain unhealed (Bagheri et al., 2020; Matoori et al., 2021; Wan et al., 2021). The essential reason for this delayed healing is that the angiogenesis process is severely impaired in a diabetic wound, which leads to the lack of sufficient support of nutrition and oxygen for wound repair (Velazquez, 2007; Okonkwo and DiPietro, 2017; Wu et al., 2019). It is believed that high glucose-induced overproduction of reactive oxygen species (ROSs) in diabetic wounds is highly associated with angiogenesis impairment since ROSs significantly damage the endothelia cells (the key cells in forming the blood vessels) (Bitar, 2019; Zhao et al., 2020; Nensat et al., 2021). Moreover, the reduction and dysfunction of endothelial progenitor cells (EPCs) is another crucial cause that leads to angiogenesis impairment as the EPCs differentiate to form the vascular endothelial cells for angiogenesis (Fadini et al., 2005; Fiorina et al., 2010; Albiero et al., 2011; Stojadinovic et al., 2014; Wang et al., 2021). According to the above analysis, we believe that attenuating the ROSs’ level as well as restoring the EPCs will be an effective strategy to efficiently restore the blood vessels for enhanced diabetic wound healing. However, to the best of our knowledge, few relevant studies have been reported so far. Therefore, in this work, we aimed to develop a novel approach to realize diabetic wound healing by combining the ROSs’ control and EPCs’ restoration.

Metformin (Met) is the most widely used prescription drug for the treatment of diabetes (Carvalho-Filho et al., 2005). Recent evidence from *in vitro* and *in vivo* studies showed that metformin may also affect the metabolic processes related to inflammation and oxidative damage (Soydas et al., 2021). Furthermore, metformin has been found to improve the impaired EPCs’ function in diabetic mice, and then stimulate angiogenesis (Yu et al., 2016; Bagheri et al., 2020). In addition, fibroblast growth factor 21 (FGF21) is an endocrine hormone derived from the liver, which has pleiotropic effects on the body and can directly improve lipid and glucose metabolism in cells (Salminen et al., 2017; BonDurant and Potthoff, 2018). A recent study revealed that FGF21 can directly act on EPCs and protect EPCs in improving diabetic angiogenesis and blood perfusion (Dai et al., 2021). More interestingly, FGF21 was found to increase the therapeutic effect of metformin (Thi et al., 2020). These studies inspired us to combine metformin and FGF21 for diabetic wound healing as they may jointly restore the EPCs’ level and functionality. So far, few reports have used this two-drug combination for diabetic wound healing.

To realize the drug combination, a drug carrier system is highly expected. Currently, the widely accepted systems for wound healing include patches (Chen et al., 2020), nanoparticles (Gan et al., 2019), and hydrogels (Wang et al., 2022). As a kind of three-dimensional porous material with high humidity, hydrogels have received especially extensive attention because of their great potential in promoting wound healing by ameliorating the wound environment (e.g., moisture, inflammation, and angiogenesis) (Kirker et al., 2002; Gong et al., 2013; Asadi et al., 2021). More importantly, by incorporating certain chemicals or structures, it will be feasible to endow the hydrogels with special functionalities to meet specific requirements for wound repair. For instance, injectable hydrogels are generally devised for wound treatment as these hydrogels can be easily and perfectly adapted to fill all wound beds without considering the wound shape (Dimatteo et al., 2018). Tissue adhesiveness is also useful for hydrogels since additional fixation can be avoided when applying them to the wound beds (Chen et al., 2018). As ROSs impede diabetic wound healing, ROS-scavenging hydrogels are developed to improve healing efficacy by introducing ROS-responsive components in the hydrogels (e.g., polyphenolic compounds and phenylborate ester) (Liu et al., 2020; Thi et al., 2020; Zhao et al., 2020). Inspired by these studies, we propose to encapsulate metformin and FGF21 in an adhesive, injectable, and ROS-scavenging hydrogel. By taking advantage of metformin and FGF21 for EPC restoration and hydrogel for ROS removal, we expect to achieve an effective therapy for diabetic wounds by this drug combination system.

Therefore, in this study, metformin and FGF21 co-loaded ROS-scavenging hydrogel with tissue adhesiveness and injectability was developed. To fabricate this hydrogel, the polyvinyl alcohol (PVA) polymer was rapidly crosslinked by a ROS-responsive crosslinker (tsPBA) in the presence of metformin and FGF21. The fast reaction between tsPBA and PVA made the hydrogel highly injectable (Cho et al., 2021). The muco-adhesiveness of PVA endowed the hydrogel with the property of tissue adhesion (Feng et al., 2021). Moreover, the existence of tsPBA enabled the hydrogel with ROS-scavenging capacity (Zhao et al., 2020). We first screened for the appropriate crosslinking ratio of tsPBA and PVA, and then measured the mechanical and ROS-scavenging properties of the hydrogel. Afterward, the *in vivo* wound healing efficacy of the hydrogel was evaluated in a rat model with a full-thickness diabetic wound. The potential mechanism of the hydrogel to accelerate diabetic wound healing was further investigated.

## Materials and methods

### Materials

Polyvinyl alcohol (PVA, Mw = 27 kDa) was purchased from Macklin (Shanghai, China). 4-Bromomethyl-phenylboric acid (BPA) and N-N-N′-N′-tetramethyl-1-3-propylenediamine (TMPA) were purchased from Aladdin (Shanghai, China). Bovine serum albumin (BSA) and 4′,6-diamidino-2-phenylindole (DAPI) were purchased from Beyotime (Shanghai, China). Hematoxylin-eosin (H&E) and Masson’s trichrome staining kits were purchased from Solarbio Science & Technology Co., Ltd. (Beijing, China). Metformin was purchased from Sigma (Shanghai, China). Fibroblast growth factor 21 (FGF21, MW = 19.4 kDa) was offered by the Key Laboratory of Biotechnology and Pharmaceutical Engineering, Wenzhou Medical University, China. All reagents used were analytic reagent (AR) grade and were used as received.

### Synthesis of reactive oxygen species-responsive crosslinker

ROS-responsive crosslinker tsPBA was synthesized according to previous reports (Wang et al., 2018). In brief, BPA (4.7 mmol) and TMPA (1.5 mmol) were dissolved in 40 ml of N, N-dimethylamide (DMF) and stirred at 60°C for 24 h. Then, the reaction mixture was poured into 100 ml of tetrahydrofuran to precipitate the product. The precipitant was collected by centrifugation and washed with THF (3 ml × 20 ml). Finally, the precipitant was dried in vacuum overnight to obtain tsPBA. The structure of tsPBA was validated by ^1^H-NMR.

### Preparation of hydrogels

The fabrication of hydrogels was based on the rapid formation of borate ester between tsPBA and PVA. For the preparation of the hydrogel without drug encapsulation, 100 μl of tsPBA solution (10 wt% in H_2_O) was added into 500 μl of PVA solution (30 wt% in H_2_O) to immediately form the hydrogel (named as PVA^tsPBA^). To fabricate the drug-loaded hydrogel, 10 μl of FGF21 (1 mg/ml), 10 μl of metformin (50 mg/ml), and 100 μl of tsPBA (10 wt% in H_2_O) were first well mixed and then added to the 500 μl of PVA solution (30 wt% in H_2_O) to quickly obtain the hydrogel (named as FGF21 + Met@Gel). The hydrogels were stored at 4°C until further application.

### Adhesive testing

The adhesion properties of the hydrogel were evaluated by a lab shear test. Porcine skin was used as the tissue model in the test. In brief, the porcine skin was first cut into a rectangle shape (2.5 cm width × 8 cm length). Then, the hydrogel PVA^tsPBA^ and the PVA polymer were sandwiched between two pieces of porcine skin with a junction contact area of 12.5 cm^2^, respectively. Afterward, the two pieces of porcine skin were loaded onto a universal testing machine (3344, Intron, United States) and pulled at a speed of 15 mm/min. The stress–strain curves were recorded, and the adhesion strength was calculated by the maximum load divided by the initial area.

### Rheology testing

The rheology test was performed on a rheometer (HAAHE MARS 40, Thermo Scientific, USA) with a parallel plate (PP50, *Φ* = 50 mm; the gap was set to 1 mm). First, a frequency-sweep from 0.1 to 100 Hz was performed under the strain of 1% to evaluate the storage modulus (G′) and loss modulus (G″) of the 30% PVA solution and PVA^tsPBA^ hydrogel. The strain sweep oscillatory tests were conducted with the shear strain ranging from 0.1 to 10% at a frequency of 10 Hz. To determine the influence of temperature, a temperature-sweep from 5 to 40°C was scanned at a strain of 1% and a frequency of 10 Hz. *n* = 6.

### Reactive oxygen species-scavenging performance testing

To assess the ROS-scavenging ability of the hydrogel, the ROS-responsive crosslinker tsPBA or the hydrogel of PVA^tsPBA^ was incubated in a freshly prepared mixture solution of H_2_O_2_ (500 μl, 1 mM), FeCl_2_ (200 μl, 0.2 mg/ml), and methylene blue (1 ml, 10 μg/ml) for 1 h. Afterward, the mixture solution was scanned at a wavelength range of 400–800 nm by a UV-Vis spectrophotometer (TU-1901, Pgeneral, China) to evaluate the ROS level (*n* = 3).

### Release of metformin and fibroblast growth factor 21 *in vitro*

In total, 600 μl PVA^tsPBA^ hydrogel containing metformin (10 μl, 50 mg/ml) and 600 μl PVA^tsPBA^ hydrogel were immersed in 3 ml PBS and shaken in an incubator shaker at a speed of 20 rpm in 37°C, respectively. At predetermined time points (0.5, 1, 1.5, 2, 3, 4, and 6 h), the supernatant from the hydrogel was collected and replaced by an equal volume of PBS. The release medium was analyzed with an ultraviolet spectrophotometer (TU-1901, Pgeneral, China) at 233 nm. In order to eliminate the effect of interference in the absorption of other components with the drug at 233 nm, the drug-free PVA^tsPBA^ was considered as a control. The absorbance of the loaded gel at each time point was subtracted from the absorbance of the hydrogel as the absorbance of metformin (Chogan et al., 2020). The concentration range of the linear standard curve was 2.5–20 μg ml^−1^, and the coefficient of determination (*R* ^2^) was 0.9999; 600 μl hydrogel containing FGF21-Cy5 (10 μl, 1 mg/ml) was immersed in 3 ml PBS and shaken in an incubator shaker at a speed of 20 rpm at 37°C. At predetermined time points (0.5, 1, 1.5, 2, 2.5, 3, 4, and 6 h), the supernatant from the hydrogel was collected and replaced by an equal volume of PBS. The release medium was analyzed with the multi-mode microplate reader (BioTek, Synergy H1, America).

### Degradation tests

PVA^tsPBA^ hydrogel (60 μl) was injected into the wounds (6 mm) of the mice and wrapped with 3M Tegaderm film (3M Health Care, Germany), and then we observed the state of the hydrogel on the wound at 0, 2, 4, 8, 10, and 24 h.

In total, 200 mg of PVA^tsPBA^ hydrogel was weighed into the transwell, and it was put into a 15-ml centrifuge tube filled with PBS (pH = 7.4), and then the opening was sealed with parafilm. We observed the remaining hydrogel in the transwell at the corresponding time and weighed it (3 parallel samples were made in this experiment).

Degradation rate = (m-m_t_)/200*100%, m_h_: mass of the remaining hydrogel, m: total mass, m_t_: mass of the transwell.

### *In vivo* wound healing

Male Sprague–Dawley (SD) rats (10–12 weeks) were obtained from the Laboratory Animals Center of Wenzhou Medical University. All animal procedures were performed following the protocol approved by the guidelines of the Animal Ethics Committee of Wenzhou Medical University. First, the rats were made to fast for 18 h, and then they were intraperitoneally injected with STZ (60 mg/kg). On days 4, 8, and 14 after STZ injection, the fasting blood sugar of the rats was measured. Rats with a blood sugar level of over 16.7 mmol/L were selected as the diabetic rats for subsequent experiments. To create the wound model, the diabetic rats were anesthetized, and the hair on the dorsal area was shaved. Afterward, four round full-thickness wounds (12 mm in diameter) were made on the central back of each rat. Then, rats were randomly divided into four groups to receive different treatments, and each wound was injected with 100 μL PBS (the control group), the free drug combination of FGF21 and Met (0.016 mg/ml FGF21 and 0.8 mg/ml Met in 100 μl PBS), PVA^tsPBA^ (16.7 μL tsPBA and 83.3 μl PVA to form the hydrogel *in situ*), or FGF21 + Met@Gel (16.7 μl drug-loaded tsPBA and 83.3 μl PVA to form the hydrogel *in situ*). After treatment, the wounds were covered with a sheet of 3M Tegaderm film (3M Health Care, Germany) and medical bandages. All treatments were repeatedly applied to the wounds for 7 days. The wounds were photographed on days 4, 8, and 14 post-treatment, and their sizes were measured using Image-Pro plus. On days 8 and 14, rats were sacrificed, and the wound tissues were harvested, fixed in 4% paraformaldehyde, embedded in paraffin, and sectioned at a thickness of 5 μm using a microtome (LEICA RM2235, Germany) for further investigation.

### Histological analysis

For histological examination, the sections were placed in an incubator with a constant temperature of 65°C for 4 h, followed by deparaffinization in xylene for 30 min and rehydrating in descending ethanol series (100, 95, and 80%) and distilled water, respectively. Subsequently, the sections were used for H&E and Masson’s trichrome staining. After staining, the sections were photographed under a light microscope (80i, Nikon, Japan).

### Immunohistochemistry staining

Tissue sections were deparaffinized and rehydrated as mentioned earlier. Then sections were placed in 3% H_2_O_2_ for 15 min to remove the endogenous enzymes and washed three times in PBS for 5 min at room temperature. Next, 50 μl trypsin was added to each sample, and the antigen was repaired in 37°C incubators for 30 min. After being washed in PBS 3 times, the sections were blocked with 5% BSA for 30 min at 37°C. Subsequently, the slides were exposed to anti-wide spectrum cytokeratin (1:200, ab9377, Abcam) primary antibody at 4°C overnight. After being rinsed with PBS for three times, the sections were incubated with secondary antibodies (1:1000, ab150077, Abcam) for 1 h at 37°C and further developed with 3,3′-diaminobenzidine tetrahydrochloride (DAB) solution. Finally, sections were counterstained with hematoxylin for 2 min, followed by 3 min in PBS to reduce the background staining. Positive staining was indicated by brown color, which can be observed under a Nikon confocal laser microscope (A1 PLUS, Nikon Tokyo, Japan).

### Immunofluorescence staining

To study the angiogenesis behavior during the healing of diabetic wounds, the key factor related to angiogenesis, alpha smooth muscle actin (α-SMA), was obtained using immunofluorescence staining. In short, samples were deparaffinized and rehydrated as mentioned earlier. Then samples were placed in 3% H_2_O_2_ for 15 min to remove the endogenous enzymes and washed three times in PBS for 5 min at room temperature. Next, 50 μl trypsin was added to each sample, and the antigen was repaired in 37°C incubators for 30 min. After being washed in PBS 3 times, the samples were blocked with 5% BSA for 30 min at 37°C. Next, the sections were incubated overnight at 4°C with anti-α-SMA primary antibody (1:50, ab5831, Abcam). After being rinsed three times with PBST (7 min, 3 times), the sections were incubated with rhodamine-conjugated goat-anti-rabbit secondary antibody and FITC-conjugated secondary antibody (1:50, Life Technologies, United States) for 1 h at 37°C. After washing with PBST three times, the cell nuclei were stained with diamidino-phenyl-indole (DAPI) for 10 min at room temperature. The tissue samples were then observed and imaged with a Nikon confocal laser microscope (A1 PLUS, Nikon, Japan).

### Western blot

Rat wound area tissue was isolated and rapidly stored at −80°C for Western blot assays. The tissue was homogenized in modified radioimmunoprecipitation assay (RIPA) buffer, which contained 10 μl/ml protease inhibitor cocktail (GE Healthcare Biosciences, United States). After centrifugation at 12,000 rpm for 10 min at 4°C, the supernatants were collected. After quantifying the aforementioned extracts, the proteins were separated on a 10% gel and transferred onto a polyvinylidene fluoride membrane (PVDF, BioRad Hercules, United States). After blocking with 5% skim milk in TBST (Tris-buffered saline with 0.1% Tween-20) for 1.5 h at room temperature, the PVDF membranes were incubated at 4°C overnight with a rabbit polyclonal antibody to Ang-1 (1:1000, AF5184, Affinity) and β-actin (1:5000, 66009, Proteintech) at 4°C overnight. After washing with TBST three times, the membrane was treated with HRP-conjugated secondary antibodies at room temperature for 1 h. The ChemiDoc XRS + Imaging System (BioRad) was used to detect the signals. ImageJ was used to measure and quantify the band densities.

### Statistical analysis

All data were expressed as mean ± standard deviations based on at least three independent experiments. Statistical significance analysis was performed between two groups using Student’s *t-test* and determined by the one-way analysis-of-variance (ANOVA) test for comparisons between multiple groups using GraphPad Prism 5 software (GraphPad Software Inc., La Jolla, United States). For all tests, *p value<0.05, **p value < 0.01, and ***p value < 0.001.

## Results and discussion

### Characterization of polyvinyl alcohol^tsPBA^ hydrogel

**FIGURE 1:**
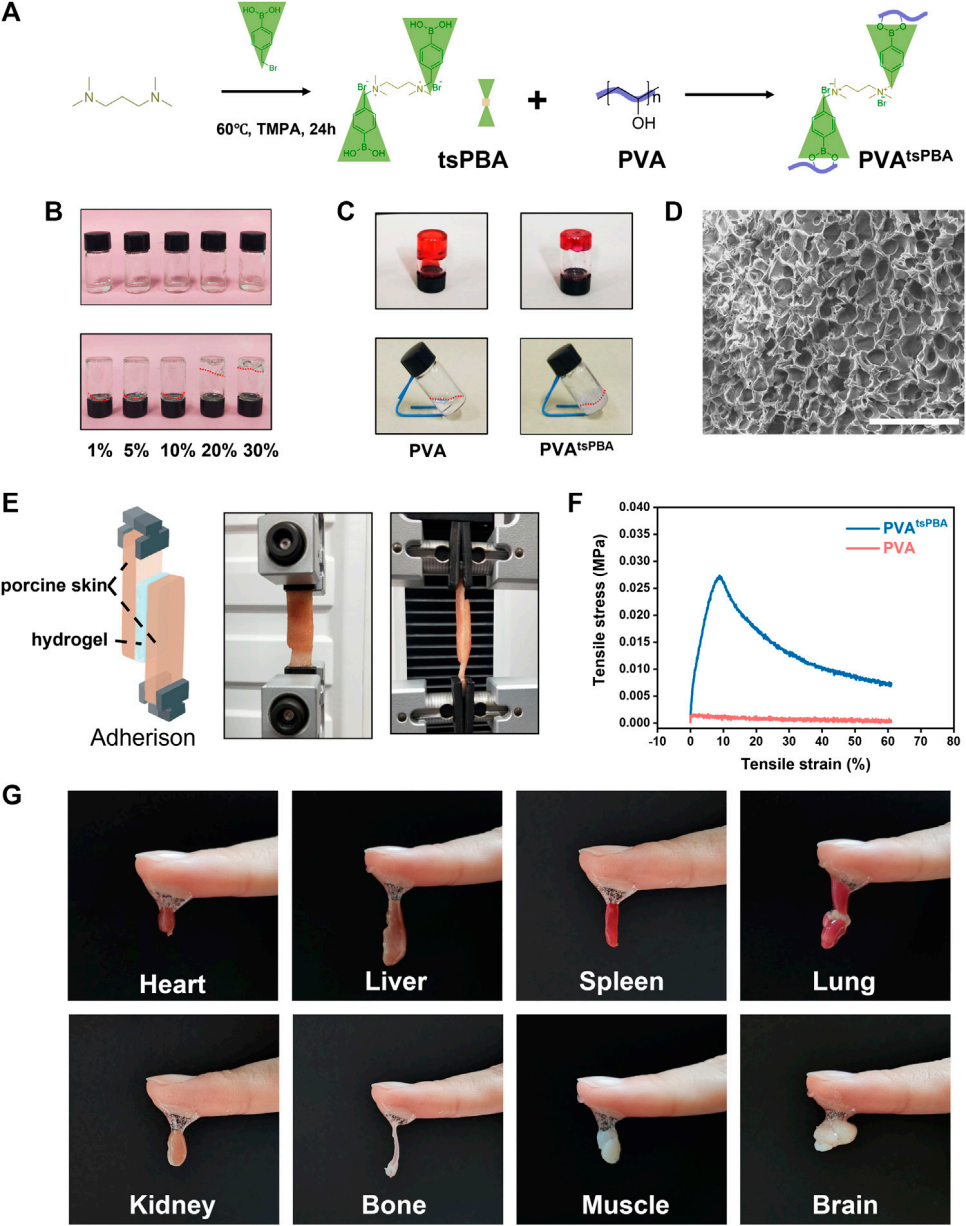
Characterization of hydrogel formed by PVA. **(A)** Simplified chemical structures of tsPBA and crosslinking with PVA. **(B)** Different physical states of the 10% tsPBA hydrogel with 1%, 5%, 10%, 20%, and 30% PVA, respectively. Prepared solutions (above). Physical state changes after several minutes (below). **(C)** 30% PVA with 10% tsPBA shows a better sol–gel transition than PVA alone. **(D)** Scanning electron microscopy (SEM) of this PVA^tsPBA^ (scale bars: 100 μm). **(E)** Representative images of PVA and PVA^tsPBA^ sandwiched between two subcutaneous fat tissues by testing the shear force using tensile mechanical test. **(F)** Adhesion strength–strain curves of the PVA and PVA^tsPBA^. **(G)** The adhesion strength of PVA^tsPBA^ to wet heart, liver, spleen, lung, kidney, bone, muscle, and brain tissues.

First, tsPBA was elaborated by a simple one-step synthesis process, and then it was crosslinked with PVA to obtain PVA^tsPBA^ as illustrated in Figure 1A. The formation of PVA^tsPBA^ was investigated through a series volume ratio of 1:5 for 10% tsPBA with 1%, 5%, 10%, 20%, and 30% PVA, respectively, as shown in Figure 1B, revealing a best sol-to-gel transition time within 1 min at room temperature for 10% tsPBA with 30% PVA. In addition, in contrast to 30% PVA itself, Figure 1C indicated that with the addition of crosslinker tsPBA, PVA^tsPBA^ formed much better hydrogel transition behavior. Scanning electron microscopy (SEM) of this PVA^tsPBA^ was further used to investigate the morphology of the hydrogel system. As expected, the hydrogel generated well-defined porous matrices as indicated in Figure 1D, which is highly desirable for wound dressing scaffolds. Moreover, as illustrated in Figure 1E, PVA^tsPBA^ and 30% PVA were, respectively, sandwiched between two subcutaneous fat tissues, and the shear force was recorded by Instron 3344, a mechanical universal test machine. The mechanical test of tensile shear stress of PVA^tsPBA^ *versus* PVA is shown in Figure 1F. The adhesive strength of PVA^tsPBA^ is about 2.16 kPa, which is significantly higher than that of PVA (0.112 kPa), indicating the higher adhesive property of PVA^tsPBA^. Eventually, we used this PVA^tsPBA^ to directly adhere to the wet tissues (indicated in Figure 1G). This ready adhesion behavior of PVA^tsPBA^ allows the hydrogel to be applied to clinical applications in a facial manner, avoiding further tedious processes to attach to the tissues. On the wound site, this good adhesive property is beneficial for the injectable gel to adhere to the irregular wound site.

**FIGURE 2:**
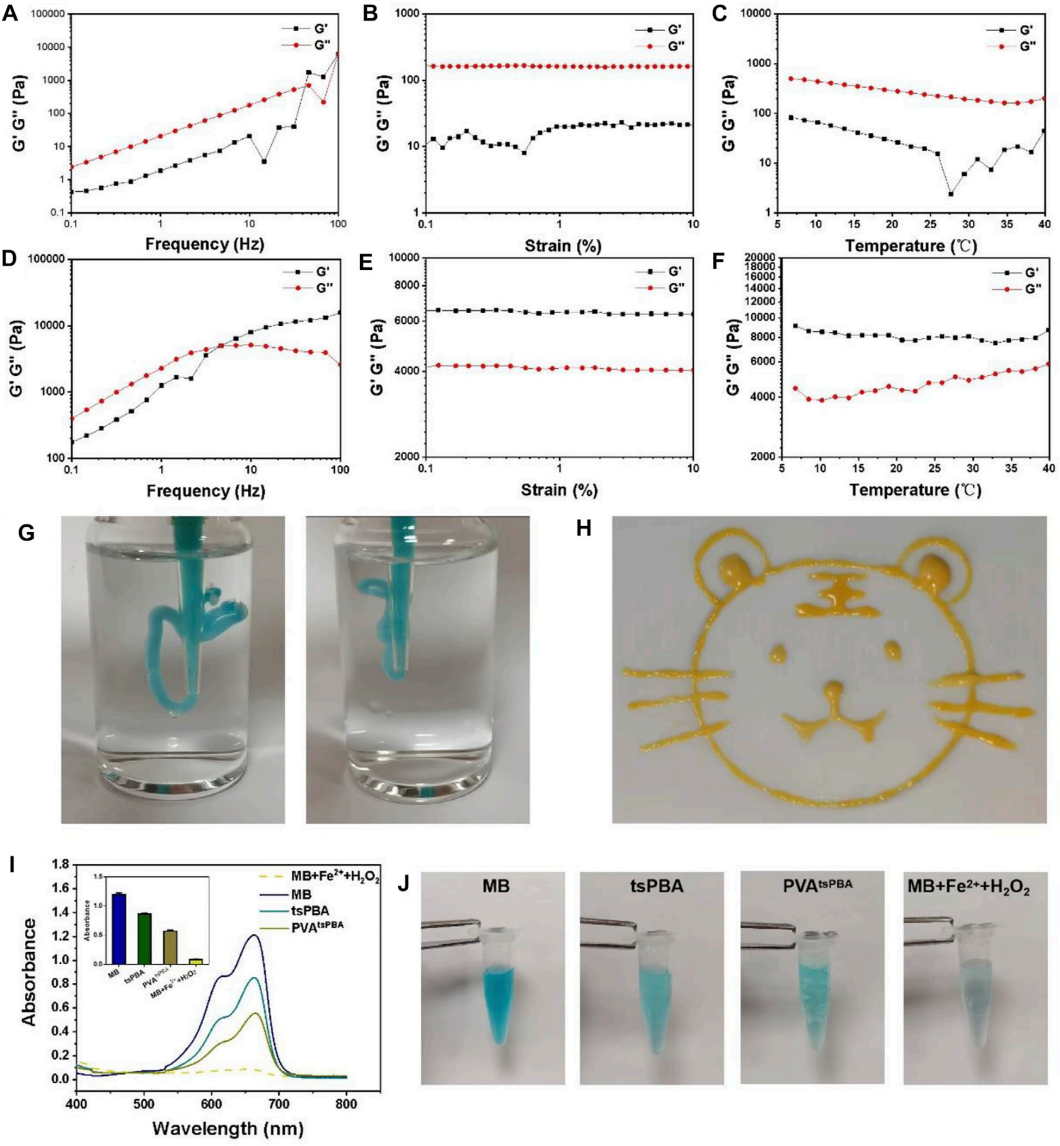
**(A)** Frequency spectra of G’ (storage modulus) and G” (loss modulus) of PVA. **(B)** Strain sweep measurements of G′ and G″ of PVA. **(C)** Evolution of the G′ and G″ over temperature for PVA. **(D)** Frequency spectra of G′ and G″ of PVA^tsPBA^. **(E)** Strain sweep measurements of G′ and G″ of PVA^tsPBA^. **(F)** Evolution of the G′ and G″ over temperature for PVA^tsPBA^. **(G)**Photograph of the injectable hydrogel. **(H)** Painting of a tiger drawn by PVA^tsPBA^ through a pipette. **(I)** UV-vis absorption spectra of MB triggered by Fenton reaction degradation with tsPBA and PVA^tsPBA^. The average of the absorbance of MB, tsPBA, PVAtsPBA, and MB + Fe^2+^+H_2_O_2_ in 663 nm *n* = 3 **(J)** The photographs of MB in Fenton reaction solution incubated with tsPBA and PVA^tsPBA^.

The rheological properties of this PVA^tsPBA^ hydrogel under various conditions were further investigated to verify the mechanical property of the hydrogel. Under the frequency range of 0.1–100 Hz, the storage modulus (G′) was significantly lower than the loss modulus (G″) for the PVA group as shown in Figure 2A, suggesting the solution state of PVA at 30% ratio. Same solution state of PVA with G′ lower than G″ were presented under shear strain from 0.1% to 10% (Figure 2B) and temperature-sweep from 5°C to 40°C (Figure 2C). However, PVA^tsPBA^ hydrogel exhibited G′ higher than G″ when shear frequency was bigger than about 4.6 Hz (Figure 2D), indicating the gel state of PVA^tsPBA^ hydrogel with the addition of crosslinker tsPBA. Moreover, the G′ was significantly higher than G″ of PVA^tsPBA^ hydrogel under both shear strain from 0.1 to 10% presented in Figure 2E and temperature from 5 to 40 °C as shown in Figure 2F, confirming the hydrogel state for PVA^tsPBA^. In addition, PVA^tsPBA^ could be extruded through the pipette (cut 200 μL pipette, Figure 2G) without clogging and be drawn into a “tiger” shape in the gel state (Figure 2H). This injectability of PVA^tsPBA^ is beneficial for the wound, especially for the irregular wound site. At the same time, PVA^tsPBA^ hydrogel also exhibited ROS-scavenging properties as the existing tsPBA. This ROS-scavenging activity was carried out by using methylene blue (MB) as the •OH indicator probe by Fenton reaction (Zhao et al., 2020). From the UV-Vis absorption spectra in Figure 2I, compared with maximum absorbance at about 650 nm of MB, the addition of ferrous ions (Fe^2+^) and H_2_O_2_ solution significantly decreases the absorbance value, indicating •OH generation, whereas the absorbance showed little decrease after adding tsPBA and PVA^tsPBA^ hydrogel, demonstrating the •OH-scavenging property of this PVA^tsPBA^ hydrogel. The blue-green color of MB change gave a much clearer compassion, as shown in Figure 2J. With the addition of Fe^2+^ and H_2_O_2_, the color of MB rapidly turned into pale blue, while the blue color stayed still for the PVA^tsPBA^ hydrogel and tsPBA-treated groups, further suggesting PVA^tsPBA^ hydrogel had an obvious ability to scavenge •OH.

### Fibroblast growth factor 21 + Met@Gel enhances diabetic wound closure

**FIGURE 3:**
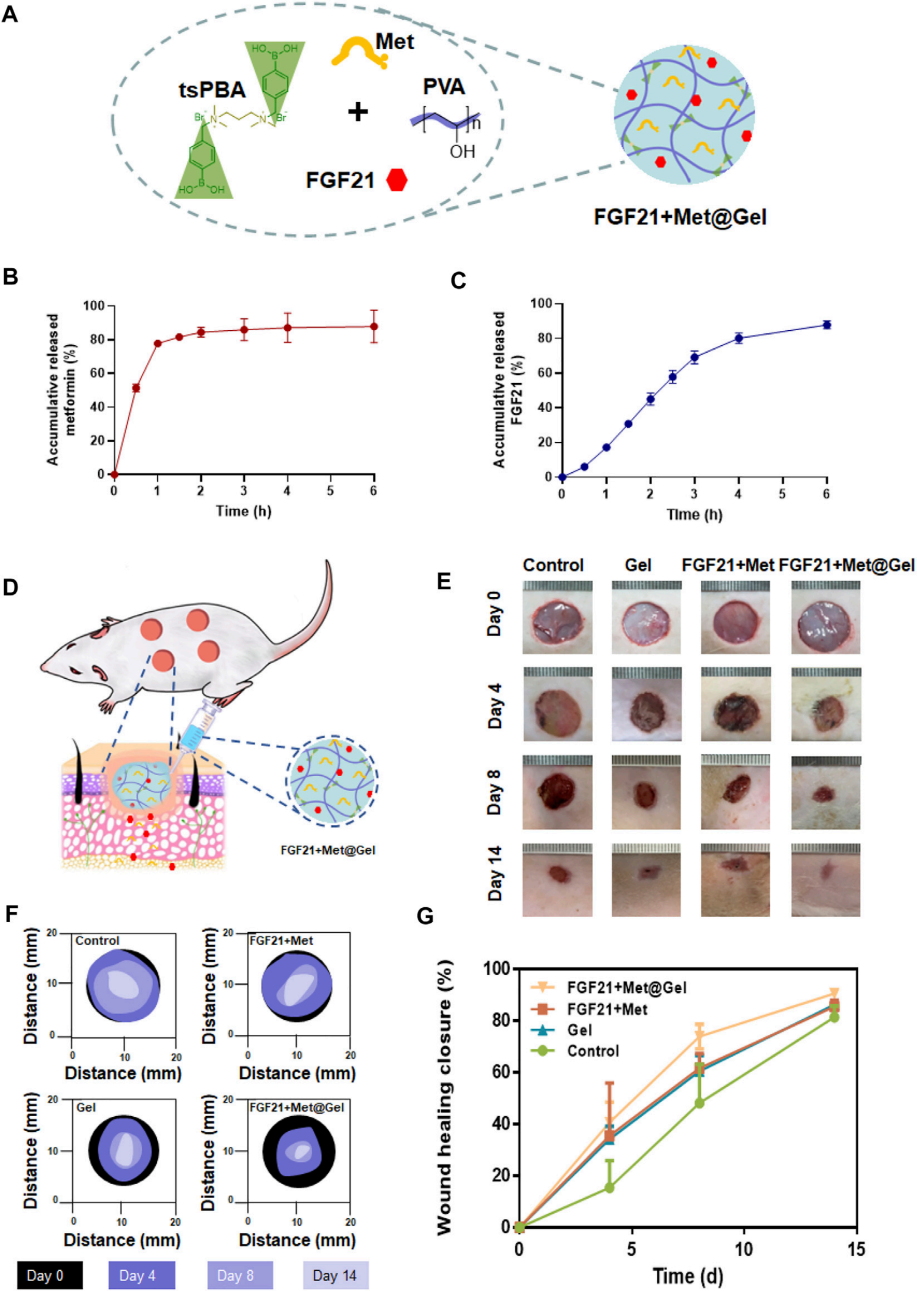
**(A)**. Schematic diagram of the drug-loaded PBA^tsPBA^ (FGF21 + Met@Gel). **(B)**. Release of metformin from PBA^tsPBA^ *in vitro, n* = 3. **(C)**. Release of FGF21 from PBA^tsPBA^ *in vitro*, *n* = 4. **(D)** Representative pictures of the rat model. **(E)** Representative images of wounds at days 0, 4, 8, and 14 after different treatments. The unit of the rulers in the images equals to 1 mm. **(F)** Wound area tracing over 14 days in each treatment category. **(G)** Curves of the wound healing closure rate *versus* treatment time. The wound closure rate is represented as a percentage of the healed wound area to the initial wound area.

Considering that both FGF21 and metformin may promote wound healing, the drugs were encapsulated in hydrogels for further experiments (Figure 3A). First, several experiments were performed to observe the release of metformin and FGF21 *in vitro*. From Figure 3B, metformin released from the hydrogel within 3 h reaches the plateau (almost 100%), indicating the successful loading of metformin. While for FGF21 in Figure 3C, FGF21 released from the hydrogel for 6 h without a plateau, suggesting the interaction of FGF21 with the hydrogel’s dedicated controlled release property. Owing to FGF21 and metformin having a positive effect on the improvement of EPC’s function in diabetic mice (Yu et al., 2016; Dai et al., 2021), we further investigated FGF21 + Met@Gel in promoting wound healing in a diabetic rat model. First, the full-thickness excisional cutaneous wounds on the dorsal skin of STZ-induced T1D SD rats were prepared to evaluate the efficacy of FGF21 + Met@Gel (Figure 3D). Diabetic wounds were treated with FGF21 + Met, single gel, FGF21 + Met@Gel, and PBS (a negative control). The wound closure efficiency was assessed by recording the macroscopic images of the wound on days 0, 4, 8, and 14, as shown in Figure 3E. Traces of the wound bed closure are presented in Figure 3F. In comparison with FGF21 + Met, single gel, and control group, wounds treated by FGF21 + Met@Gel showed the smallest wound area especially from day 8, suggesting the best wound healing efficacy of FGF21 + Met@Gel. In addition, to quantify the wound repair efficiency, the relative wound area is also statistically summarized in Figure 3G based on the captured wound images on days 0, 4, 8, and 14, revealing that FGF21 + Met@Gel significantly accelerated the wound repair with the highest wound closure rate. Especially, on day 14, the average wound healing closure rate of FGF21 + Met@Gel had already increased to 90.7%, which is remarkably higher than that of FGF21 + Met (85.5%), single gel (86.3%), and PBS (81.5%), indicating the positive effect of the FGF21 + Met@Gel systems upon the diabetic wound.

### Fibroblast growth factor 21 + Met@Gel improves micro-granulation formation and collagen deposition

**FIGURE 4:**
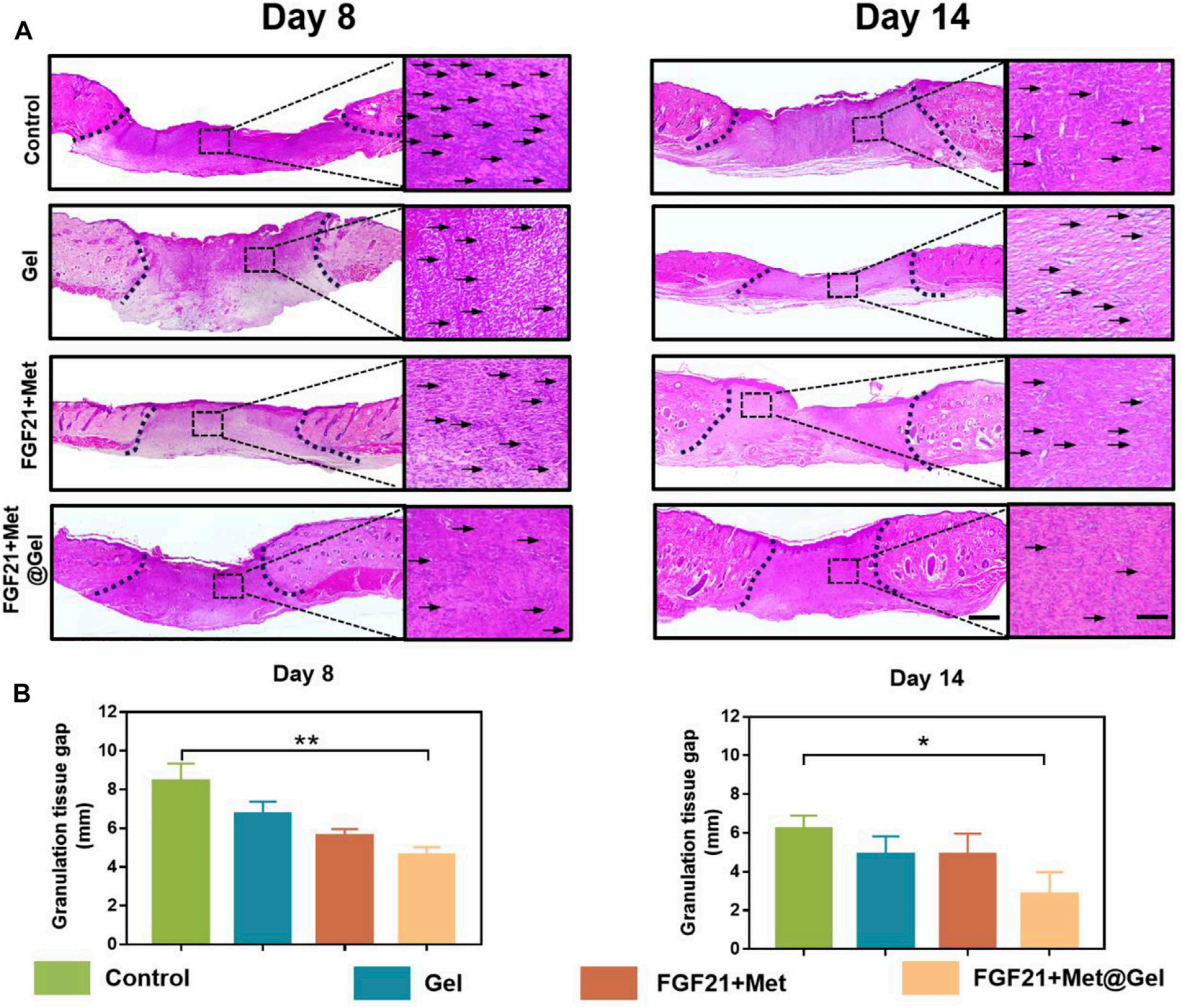
**(A)** Representative images of H&E staining of skin tissues on days 8 and 14. The scale bars are 1000 μm (left) and 250 μm (right, magnified images). Dash lines represent granulation tissue gap and arrows indicated the inflammatory cells. **(B)** Statistical analysis of granulation tissue gap based on the H&E staining. ^∗^ *p* value<0.05, ^∗∗^ *p* value < 0.01.

To further assess the effect of FGF21 + Met@Gel upon wound healing, histological analysis was performed using hematoxylin and eosin (H&E) staining to analyze the detailed granulation formation of the skin tissue sections on wounds treated with PBS (control), FGF21 + Met, single gel, and FGF21 + Met@Gel. As shown in Figure 4A, the representative histological micrographs of wound tissue sections on days 8 and 14 revealed the efficiency of wound healing after the different treatments. Based on the observation of their granulation tissue formation (area between dash lines), the FGF21 + Met@Gel-treated group exhibited the best wound regeneration behavior by improved new granulation deposition with lowest inflammatory infiltration (indicated by arrows of enlarged micrographs). Quantitative results of the regenerated granulation tissue gap on days 8 and 14 were further collected in Figure 4B, respectively. Especially, on day 14, the control (6.21 ± 0.56 mm) wounds exhibited the longest granulation tissue in the wound center, followed by Gel (4.88 ± 0.67 mm) and FGF21 + Met (4.87 ± 0.89 mm), while FGF21 + Met@Gel applied wounds were apparently the shortest (2.84 ± 0.93 mm) with much thicker and organized granulation deposition. In addition, the FGF21 + Met@Gel group also developed well-formed integral epithelium and matured hair follicles with adjoining sebaceous glands within the wound bed. To sum up, FGF21 + Met@Gel helped in diabetic wound healing by accelerating granulation tissue generation, in accordance with the previous wound closure rate, suggesting the successful design of co-delivery of FGF21 with metformin using this injectable PVA^tsPBA^ hydrogel.

**FIGURE 5:**
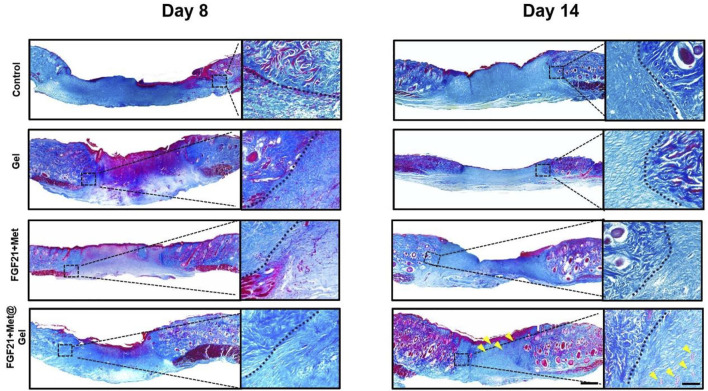
Representative images of Masson’s trichrome staining of skin tissues on days 8 and 14 (yellow arrows indicated the new regenerated follicles). The scale bars are 1000 μm (left) and 250 μm (right, magnified images).

Well-organized collagen deposition could result in better wound repair as well as enhanced skin tensile strength (Mathew-Steiner et al., 2021). Here, we investigated the newly formed collagen expressed in wounds upon different treatments through Masson’s trichrome staining. As indicated by Figure 5, it was clearly displayed that the control and FGF21 + Met-treated groups had limited distribution of blue color on day 8, revealing that few collagens were produced in the wound sites. Compared to these low levels of rare and loose collagen deposited in the wound site by the control and FGF21 + Met groups on day 8, relatively denser and well-organized collagen with darker blue color appeared in Gel and FGF21 + Met@Gel-treated wounds. The FGF21 + Met@Gel-treated wound exhibited the most abundant collagen deposition, suggesting the positive effect in improving collagen expressions in the early wound healing stage by the FGF21 + Met@Gel system. Eventually, on day 14, each group exhibited denser collagen deposition with intact upper epidermis during the increased healing time. Particularly, FGF21 + Met@Gel-treated wounds on day 14 showed the most mature and well-organized collagen with accessory hair follicles (see yellow arrows indicated in Figure 5) regenerated in the dermis, which is consistent with H&E results. Masson’s trichrome staining results further suggested that with the application of FGF21 + Met@Gel, the collagen formation in the main wound matrix was enhanced in the wound site, resulting in the improved healing efficiency and complete regeneration of the full-thickness diabetic wounds.

### Fibroblast growth factor 21 + Met@Gel activates the re-epithelization

**FIGURE 6:**
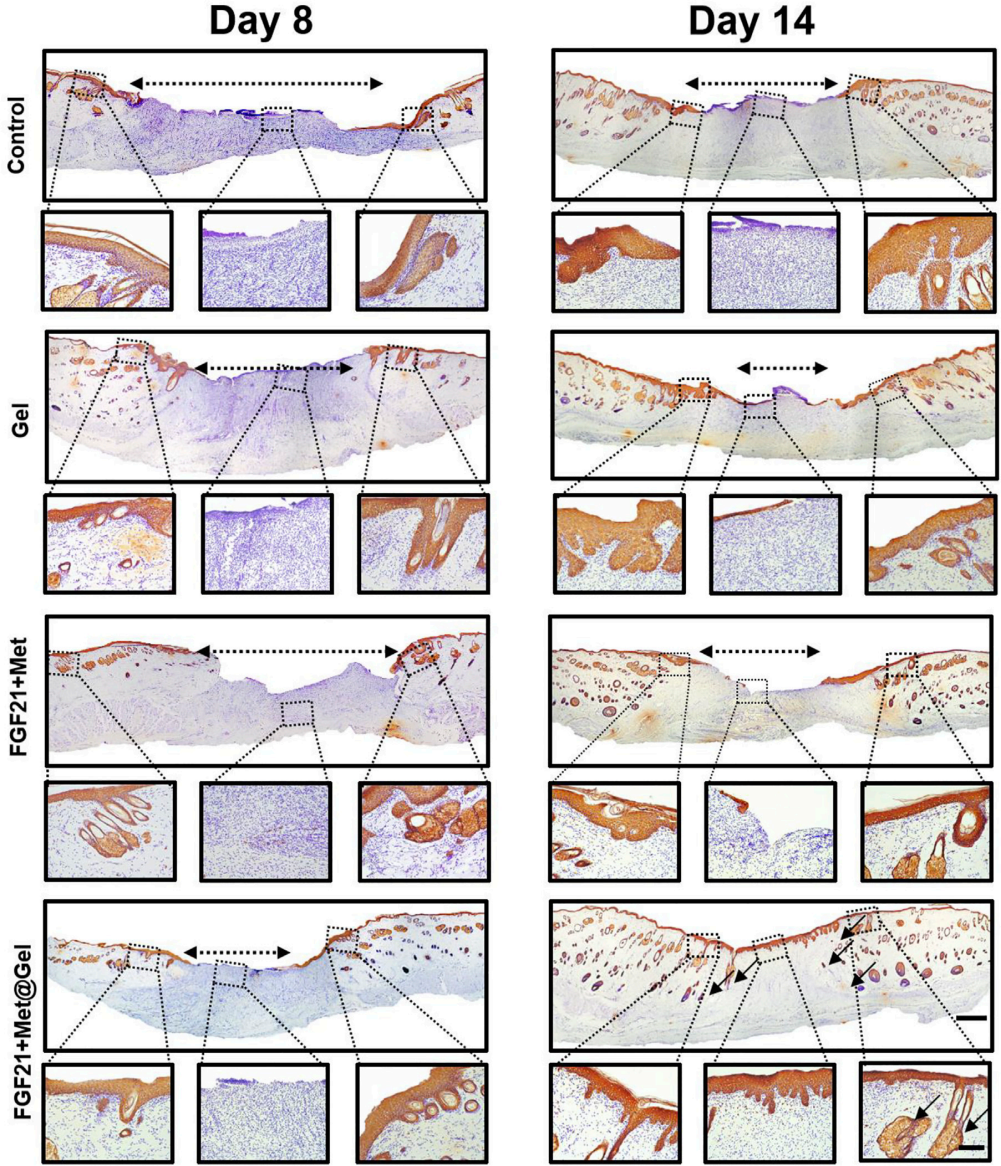
Representative images of immunohistochemical staining of skin tissues on days 8 and 14 (original scale bar = 1000 μm). Dashed box presents the corresponding higher magnification immunohistochemical staining of wound bed centers and edge (close-up scale bar = 250 μm; dash lined with double arrows indicated re-epithelization gap, arrows indicated new hair follicles).

Impaired wound healing of diabetic wounds is usually caused by a variety of diabetic complications such as delayed re-epithelization and deficient angiogenesis (Zhang et al., 2015; Wang et al., 2019). Re-epithelialization often refers to the formation of new epithelium, which can be stained with wide-spectrum cytokeratin (Wu et al., 2016). Here, to further detect the positive effect of the drug-loaded gel upon diabetic epithelium recovering, cytokeratin immunohistochemical staining of the wound tissues was used after different treatments on days 8 and 14. As shown in Figure 6, on day 8, the control, FGF21 + Met, and single gel groups all had large unhealed re-epithelium, while FGF21 + Met@Gel exhibited an obvious smaller epidermal gap (dash lines with double-headed arrows), indicating the enhanced crawling speed of the epidermis of FGF21 + Met@Gel group. Moreover, on day 14, compared to the incomplete epidermis of the wound (dash lines with double-headed arrows) treated with control, FGF21 + Met, and single gel groups, FGF21 + Met@Gel applied wound showed an integrated continuous uniform epidermis layer covering the wound with newly regenerated hair follicles (black arrows) in the wound site. This result indicated that FGF21 + Met@Gel achieved complete epithelialization on day 14 by accelerating the migration of the epithelial cells. The enhanced degrees of re-epithelialization further confirmed that FGF21 + Met@Gel could stimulate wound regeneration similar to H&E and Masson’s staining.

### Fibroblast growth factor 21 + Met@Gel stimulates the angiogenesis by recruiting endothelial progenitor cells *via* upregulation of Ang-1

**FIGURE 7:**
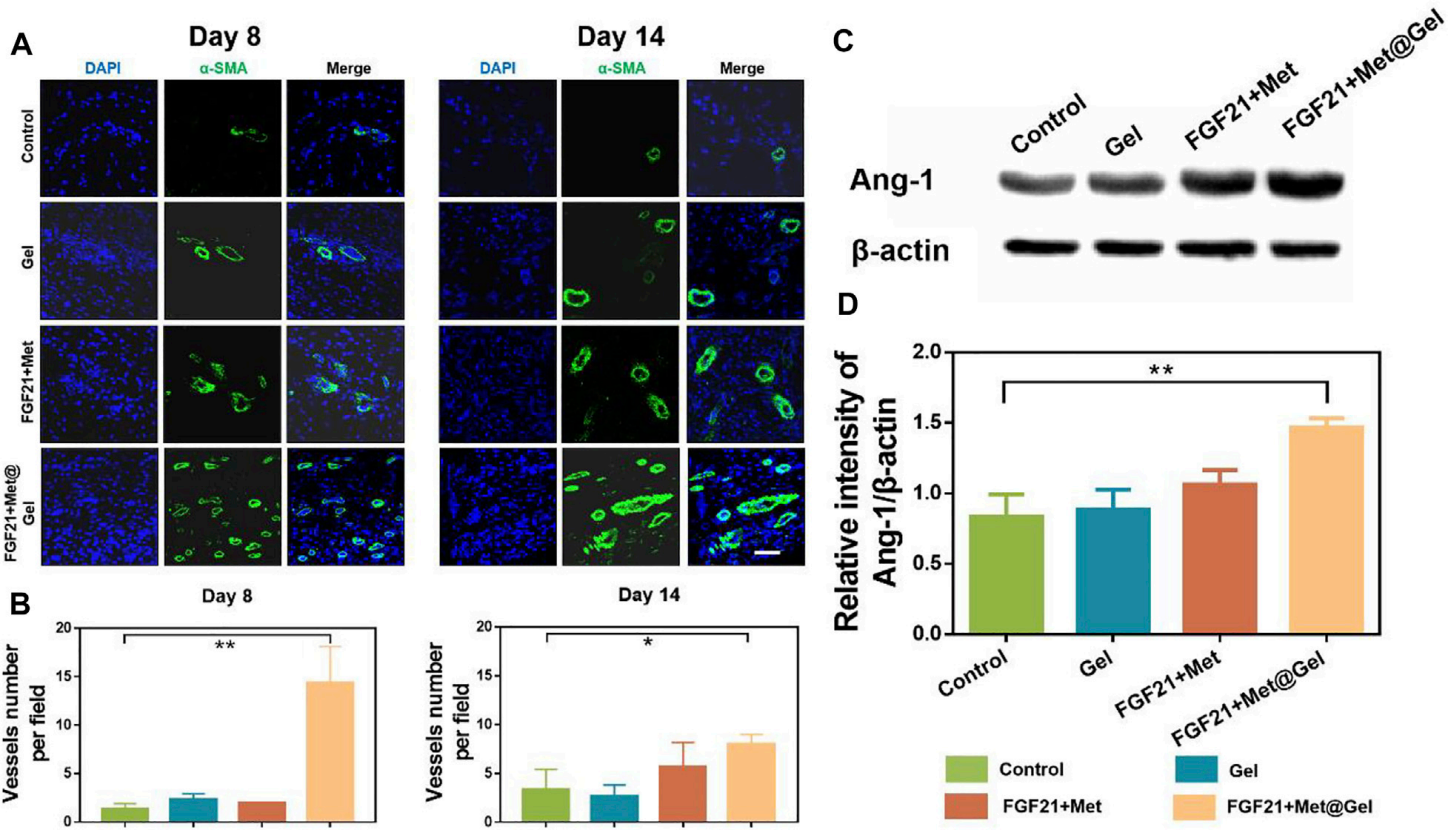
**(A)** Immunofluorescence staining of α-SMA (green) of wound tissues on days 8 and 14 (scale bars: 50 μm). **(B)** Quantified data of vessels number per field. **(C)** Western blot analysis of Ang-1 expression. **(D)** Quantitative analysis of Ang-1 protein expression based on Western blot. *p < 0.05; **p < 0.01 compared with control, *n* = 3.

In the process of wound repair, regeneration blood vessels help provide oxygen for cell proliferation and migration, which contributes to wound healing eventually. Diabetic wounds failed with active vascular remodeling due to complicated micro-environment and impaired functionalized EPCs. Thus, the degree of vessel remodeling, vessel integrity, and maturation of the wound are crucial factors to evaluating diabetic wound healing efficiency. Herein, Figure 7A presents the immunofluorescent staining of the endothelial marker α-SMA on days 8 and 14 of wound tissues after different treatments. Compared to the rare positive fluorescent blood vessels of the control group on both days 8 and 14, gel, FGF21 + Met, and FGF21 + Met@Gel all increased the expression of positive green fluorescence, suggesting the stimulation of neovascularization by the drug and the gel during the wound healing process. Among all the groups, the positive green fluorescence of FGF21 + Met@Gel showed significant enhancement of the positive newly formed mature blood vessels, revealing that FGF21 + Met@Gel accelerated the regeneration and maturation of blood vessels to accommodate the urgent demand for oxygen and nutrients for wound regeneration. In addition, quantitative analysis of the density of blood vessels as shown in Figure 7B confirmed that FGF21 + Met@Gel had the best rebuilding property in blood vessels (*p < 0.05). These data suggest that FGF21 + Met@Gel significantly promoted diabetic wound angiogenesis, which ultimately led to rapid wound repair.

Next, we would like to know whether the enhanced angiogenesis in wounds treated by FGF21 + Met@Gel was related to the restoration of EPCs. As EPCs were recruited by Angiopoietin-1 (Ang-1) in diabetic wounds (Lemieux et al., 2005; Balaji et al., 2015), the expression level of Ang-1 could be used to reflect the EPCs’ level. Therefore, Ang-1 expressions in wounds treated by different groups were measured by Western blot as shown in Figures 7C,D. Compared with Ang-1 expressions in the control group, both Gel and FGF21 + Met-treated groups exhibited a slighter increase in Ang-1 expressions of 1.04-fold and 1.27-fold, respectively, while FGF21 + Met@Gel caused a more obvious enhancement of Ang-1 expression by 1.75-fold (**p < 0.01). This result confirmed that FGF21 + Met@Gel triggered the upregulation of Ang-1 and led to the recruitment of EPCs, which eventually improved angiogenesis in diabetic wounds. Thus, all the above data proved that FGF21 + Met@Gel stimulated angiogenesis through EPC recruitment to the diabetic wound, further accelerating wound healing.

## Conclusion

In conclusion, in this study, we designed a novel PVA^tsPBA^ hydrogel system by loading biological macromolecule glucose/lipid metabolic regulator FGF21 and metformin to accelerate diabetic wound regeneration. The PVA^tsPBA^ hydrogel exhibited suitable adhesive property, *in situ* injectability, proper rheological, and ROS-scavenging ability that could be affiliated to the microenvironment of diabetic wounds to promote wound healing. Finally, the therapeutic effect of FGF21 + Met@Gel hydrogel on wound regeneration was investigated using a full-thickness skin defect diabetic rat model. Compared with the control, gel, and only FGF21 + Met groups, this FGF21 + Met@Gel significantly accelerated the process of wound repair in the wound closure rate, granulation formation, collagen deposition, and re-epithelization of diabetic wounds. Moreover, further immunofluorescence staining as well as Western blot evaluation indicated that FGF21 + Met@Gel stimulates the angiogenesis of diabetic wounds by recruiting EPCs *via* upregulation of Ang-1 expression in the wound. All of these results suggest that this adhesive *in situ* FGF21 + Met@Gel is an excellent candidate as a bioactive wound dressing for diabetic cutaneous wound healing. This work provides a novel approach for delivering biological macromolecules FGF21 with collaborative delivery of chemical compound metformin to the wound site using a novel hydrogel system to promote wound regeneration.

## Data Availability

The original contributions presented in the study are included in the article/Supplementary Material; further inquiries can be directed to the corresponding authors.
